# Implementation of and Early Outcomes From Anal Cancer Screening at a Community-Engaged Health Care Facility Providing Care to Nigerian Men Who Have Sex With Men

**DOI:** 10.1200/JGO.19.00102

**Published:** 2019-07-19

**Authors:** Rebecca G. Nowak, Nicaise Ndembi, Wuese Dauda, Paul Jibrin, Søren M. Bentzen, Chinedu H. Nnaji, Oluwole Olaomi, Teresa M. Darragh, Jonathan Madukwe, Trevor A. Crowell, Stefan D. Baral, William A. Blattner, Manhattan E. Charurat, Joel M. Palefsky, Kevin J. Cullen, on behalf of the TRUST/RV368 Study Group

**Affiliations:** ^1^ University of Maryland School of Medicine, Baltimore, MD; ^2^ Institute of Human Virology Nigeria, Abuja, Nigeria; ^3^ National Hospital, Abuja, Nigeria; ^4^ University of California, San Francisco, CA; ^5^ Walter Reed Army Institute of Research, Silver Spring, MD; ^6^ Henry M. Jackson Foundation for the Advancement of Military Medicine, Bethesda, MD; ^7^ Johns Hopkins Bloomberg School of Public Health, Baltimore, MD

## Abstract

**PURPOSE:**

Anal cancer risk is substantially higher among HIV-infected men who have sex with men (MSM) as compared with other reproductive-age adults, but screening is rare across sub-Saharan Africa. We report the use of high-resolution anoscopy (HRA) as a first-line screening tool and the resulting early outcomes among MSM in Abuja, Nigeria.

**METHODS:**

From August 2016 to August 2017, 424 MSM enrolled in an anal cancer screening substudy of TRUST/RV368, a combined HIV prevention and treatment cohort. HRA-directed biopsies were diagnosed by histology, and ablative treatment was offered for high-grade squamous intraepithelial lesions (HSIL). HRA proficiency was assessed by evaluating the detection of squamous intraepithelial lesions (SIL) over time and the proportion biopsied. Prevalence estimates of low-grade squamous intraepithelial lesions and HSIL with 95% CIs were calculated. Multinomial logistic regression was used to identify those at the highest risk of SIL.

**RESULTS:**

Median age was 25 years (interquartile range [IQR], 22-29), median time since sexual debut was 8 years (IQR, 4-12), and 59% (95% CI, 54.2% to 63.6%) were HIV infected. Rate of detection of any SIL stabilized after 200 screenings, and less than 20% had two or more biopsies. Preliminary prevalence estimates of low-grade squamous intraepithelial lesions and HSIL were 50.0% (95% CI, 44.7% to 55.3%) and 6.3% (95% CI, 4.0% to 9.3%). HIV infection, at least 8 years since anal coital debut, concurrency, and external warts were independently statistically associated with SIL.

**CONCLUSION:**

Proficiency with HRA increased with experience over time. However, HSIL detection rates were low, potentially affected by obstructed views from internal warts and low biopsy rates, highlighting the need for ongoing evaluation and mentoring to validate this finding. HRA is a feasible first-line screening tool at an MSM-friendly health care facility. Years since anal coital debut and external warts could prioritize screening.

## INTRODUCTION

Anal cancer risk is 52-fold higher in HIV-infected men who have sex with men (MSM) in the United States compared with the general population,^1^ and anal high-grade squamous intraepithelial lesions (HSIL) are more likely to progress than to regress with HIV coinfection.^2^ The syndemic of HIV and high-risk human papillomavirus (HR-HPV) drives the increasing incidence of anal cancer among MSM in the United States.^1^ We have reported previously, among MSM receiving care at a community-engaged health care facility in Abuja, Nigeria, an HIV prevalence of 45% and a HR-HPV prevalence of 90% for those living with HIV.^3,4^ As HIV-infected MSM in sub-Saharan Africa age with the use of effective antiretroviral therapy, an increasing anal cancer incidence can be expected, paralleling current trends in the United States.^1^

**Context**

**Key Objective**
Could high-resolution anoscopy (HRA) be used as a first-line screening tool for men at high risk of anal cancer across sub-Saharan Africa?
**Knowledge Generated**
HRA screening was highly attended, with more than 500 screenings among men who have sex with men (MSM) in the context of a culturally and clinically competent health care facility providing HIV and sexually transmitted infection care in Nigeria. HRA clinical proficiency improved with experience, although preliminary estimates of high-grade squamous intraepithelial lesions were low, suggesting the need for ongoing evaluation and clinical mentorship. MSM with anal warts and at least 8 years since anal coital debut were at highest risk of high-grade squamous intraepithelial lesions; therefore, these are potential indications for priority screening.
**Relevance**
With sufficient mentorship, HRA represents an effective secondary cancer prevention approach for MSM even in the context of challenging social environments, suggesting the need for broader evaluation of implementation strategies.

High-resolution anoscopy (HRA) is a sensitive diagnostic test but is generally used as a second-line screening tool after a positive anal Pap test because of its steep learning curve.^5-8^ In Nigeria, however, liquid cytology is not available, and many factors would hinder accurate and timely diagnosis; therefore, we deferred to HRA as the most sensitive and cost-effective method.^8,9^ A colposcope helps the examiner visualize biopsy areas suspected to be HSIL or cancer in the anal canal and the perianal areas. Ablative therapy is used to treat biopsy-proven anal HSIL, extrapolating from data showing that treatment of cervical HSIL in women reduces the incidence of cervical cancer. Challenges with mastering HRA include understanding the topographic anatomy of the anal canal and working in a three-dimensional space with good eye-hand coordination.^5^ Lesions may be obscured by internal warts, hemorrhoids, or folds.^5^ HRA also requires a coordinated team of specialists to optimize care, including a skilled pathologist and a surgeon capable of performing excisional biopsies, treating extensive HSIL, and managing complicated cases.^5^ Despite these challenges, we decided to assess the feasibility of HRA in a low-resource setting with high rates of HIV and HR-HPV.

Even with specialized training in HRA and a skilled interdisciplinary team, the success of a screening program may be further complicated by cultural environments in which same-sex practices are stigmatized or criminalized. Nigerian MSM face high levels of discrimination and must be cautious of environments in which their sexual identity could be disclosed.^10,11^ Homophobia is also problematic in Nigerian health care settings; nearly a quarter of medical students in one study believed MSM should be denied health care services.^12^ The objective of this study was to evaluate participant acceptability of and clinician proficiency with using HRA as a first-line screening tool at an MSM-friendly community-engaged health care facility in Nigeria. Secondary objectives included evaluating the prevalence and correlates of anal squamous intraepithelial lesions (SIL).

## METHODS

### Training and Mentoring

Two Nigerian physicians, one trained in infectious diseases and the other in surgery, attended a 4-day comprehensive colposcopy and high-resolution anoscopy training course conducted by the American Society for Colposcopy and Cervical Pathology in Providence, RI. After the course, the infectious disease physician shadowed experienced HRA practitioners for 4 days at the Anal Neoplasia Clinic, Research and Education Center at the University of California, San Francisco (UCSF) Helen Diller Family Comprehensive Cancer Center. A Nigerian pathologist also traveled to San Francisco to receive mentoring on anal cytologic and histologic samples at the UCSF Department of Pathology. UCSF physicians were available throughout the study for guidance, and one conducted 2 week-long, on-site mentoring sessions in Nigeria, including one focused on screening techniques 3 months after study initiation and a second on treatment-oriented session at study closure.

### Study Design and Population

Anal cancer screening was performed in Abuja, Nigeria, as a substudy of the previously described TRUST/RV368 cohort study.^13,14^ In brief, TRUST/RV368 recruits MSM using respondent-driven sampling into comprehensive HIV and sexually transmitted infection prevention, treatment, and care. Between August 2016 and August 2017, participants of TRUST/RV368 who were 18 years of age or older were educated on the rationale and procedures of the screening study. Interested men were enrolled if they provided separate informed consent in English or Hausa. Exclusion criteria included an allergy to lidocaine or iodine, as well as any medical condition that could increase the risk associated with HRA or anal biopsy, such as a bleeding disorder.

The anal cancer screening substudy included a training period, during which the Nigerian physician performed at least 100 HRAs before additional on-site mentoring by UCSF staff. Prior data have suggested that this number of HRAs is sufficient to achieve proficiency.^15^ Once training was complete, at least 350 men were screened to estimate the prevalence and correlates of SIL. Men seen during training were allowed to be rescreened.

### Clinical Procedures

All participants completed a questionnaire that captured data on smoking, anal cancer symptoms, and sexual behaviors. Additional clinical and behavioral data were obtained from the parent TRUST/RV368 study. Participants underwent digital anorectal examination, swabbing of the anal canal for future HPV testing, HRA, and HRA-directed biopsies of abnormalities for histologic confirmation. Cytology slides were introduced as a quality control measure after training to capture any missed HSIL. Liquid-based cytology was unavailable, so anal swabs were smeared on slides and fixed in 100% alcohol. Cytology slides were classified using the 2001 Bethesda System, and cells were interpreted as negative, atypical squamous cells of undetermined significance, atypical squamous cells, cannot exclude HSIL, low-grade squamous intraepithelial lesions (LSIL), or HSIL.^16^

During HRA, 5% acetic acid and Lugol’s iodine were applied to the squamocolumnar junction to visualize abnormalities. Only men with acetowhite lesions underwent biopsies. The biopsy specimens were fixed in formalin and embedded in paraffin before processing for routine hematoxylin and eosin histopathologic assessment. Biopsy specimens were classified according to the Lower Anogenital Squamous Terminology^17^ as benign, low-grade squamous intraepithelial neoplasia (LSIL), HSIL, or squamous cell carcinoma. Ablative therapy with hyfrecation was offered for those with biopsy-diagnosed HSIL.

After study completion, UCSF reviewed 21 histologic samples representing benign to HSIL. Percent agreement and unweighted Kappa were calculated to compare consistency in histology diagnosis between the two institutions.

### Ethical Considerations

The institutional review board at the Nigerian Federal Capital Territory Health Research Ethics Committee, the Clinical Research Committee at the University of Maryland Marlene and the Stewart Greenebaum Comprehensive Cancer Center, the University of Maryland Baltimore, and the University of California San Francisco institutional review board reviewed and approved the research protocol.

### Statistical Analyses

The prevalence of detected anal SIL and the proportion biopsied between training and screening were used as metrics to assess proficiency with HRA. Any differences in participants between training and screening were evaluated using Pearson’s χ^2^ and Fisher’s exact tests. HRA proficiency was expected to be achieved after performing the procedure on at least 100 men.^15^ Stabilization of the prevalence of LSIL and HSIL was used as an indicator of proficiency^6^; to this end, prevalence of any SIL and 95% CIs were estimated for 12 consecutive intervals throughout the year. Each interval was made up of 43 to 44 men. The prevalence and 95% CIs were plotted at the midpoint of each interval and fitted to a standard growth curve to approximate a learning curve.

The primary outcome of screening was a multinomial categorization of anal dysplasia: benign, LSIL, or HSIL. Benign outcomes included those who did not undergo a biopsy during HRA because no lesions were seen. Histology determined the final diagnosis during training. During screening, a composite diagnosis was generated from the worst grade of either cytology or histology. Cytologic diagnoses of atypical squamous cells of undetermined significance were categorized as negative/benign.

Data collected after training were used to evaluate predictors of anal SIL. Demographic characteristics included age, education, marital status, sexual orientation, and HIV status. Behavioral characteristics included smoking, preferred sexual position (anal insertive, anal receptive, or both), years since anal coital debut, lifetime number of receptive partnerships, transactional sex, and concurrency. Transactional sex was defined as having exchanged anal or oral sex for things wanted or needed such as money, drugs, food, shelter, or transportation.^18^ Concurrency was defined as having multiple sex partners at the same time in the past year (both men and women). Clinical characteristics included self-reported swollen or tender lymph nodes around the groin area, any hardening or narrowing of the anal passage or stenosis, anal herpes, and external anal warts at the time of HRA. Nucleic acid amplification testing was performed at the visit before HRA to diagnose anorectal or urethral *Neisseria gonorrhea* and/or *Chlamydia trachomatis*.

Crude differences in participant characteristics by anal SIL during screening were compared using Pearson’s χ^2^ and Fisher’s exact tests. Bivariate and multivariable multinomial logistic regression was used to estimate odds ratios and 95% CIs. All variables associated with anal SIL in bivariate analysis (*P* ≤ .10) were included in the multivariable model. Variables that were insignificant (*P* ≥ .05) were removed in a backward stepwise approach to obtain the most efficient model. Lifetime number of receptive partners and sexual position were not retained because of strong correlations with years since anal coital debut and external anal warts. Observations with missing covariates accounted for less than 5% of the total sample and were retained in the multivariable analysis. Data were analyzed using Stata Statistical Software, Release 13 (StataCorp, College Station, TX).

## RESULTS

**FIG 1:**
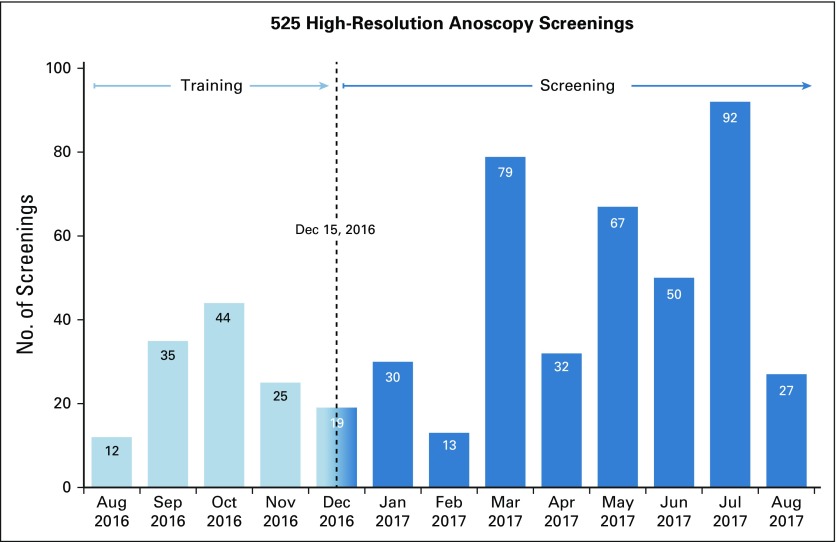
High-resolution anoscopy screenings by month.

Of 444 men eligible for screening, 424 (95%) participated. The median age was 25 years (interquartile range [IQR], 22-29 years), and the median time since anal coital debut was 8 years (IQR, 4-12 years). Nearly 60% (250 of 424) were HIV infected, with a median CD4 of 464 count/mm^3^ (IQR, 299-626 count/mm^3^) and a median HIV RNA viral load of 27 copies/mL (IQR, 20-7,946 copies/mL). Fifty-eight participated in training alone, 302 participated in screening alone, and 64 participated in both. For men multiply screened, only the most recent diagnosis was included in the modeling. In total, 525 screenings were conducted (Fig 1), with five missing histology diagnoses.

**FIG 2:**
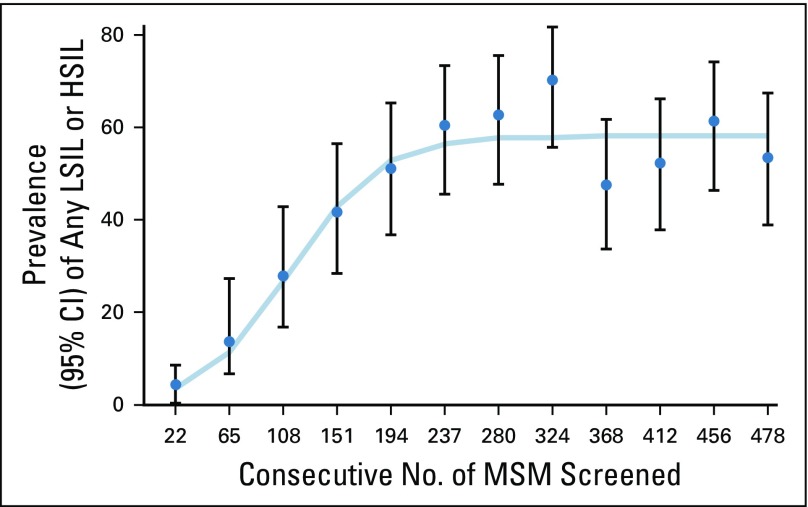
Detection of any low-grade squamous intraepithelial lesions (LSIL) or high-grade squamous intraepithelial lesions (HSIL) over time (August 2016 to August 2017). MSM, men who have sex with men.

**FIG 3:**
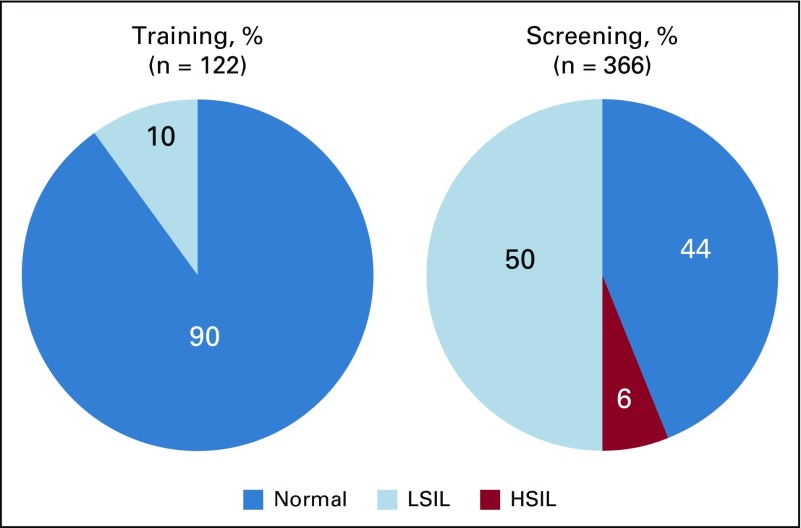
Anal dysplasia during training and screening. HSIL, high-grade squamous intraepithelial lesions; LSIL, low-grade squamous intraepithelial lesions.

Compared with the men screened, those during training were more likely to be HIV infected, had more receptive sex partners, had more concurrent partnerships with strictly men, and were more likely to self-report anal herpes, but they were less likely to have external warts (Table 1). Only 15% of men during training underwent at least one biopsy compared with 51% of men during screening. Diagnosis for any SIL grew linearly for the first 200 screenings, and prevalence estimates stabilized for the remainder of the study (Fig 2). The prevalence of LSIL was 10.1% (95% CI, 5.3% to 17.0%) during training and 50.0% (95% CI, 44.7% to 55.3%) during screening. HSIL was not diagnosed during training and was 6.3% (95% CI, 4.0% to 9.3%) during screening (Fig 3). HSIL was detected more among HIV-infected than among HIV-uninfected men (8% *v* 4%, *P* < .01). One case of HSIL was diagnosed by cytology and the rest by histology. Agreement in the histology diagnoses between UCSF and Nigeria was 71% (Kappa = 0.56; 95% CI, 0.26 to 0.86), suggesting moderate agreement. The histologic diagnosis was not altered if discrepant with UCSF to avoid introducing bias. Sixty-one percent (14 of 23) of the cases of HSIL returned for ablative treatment.

**TABLE 1:**
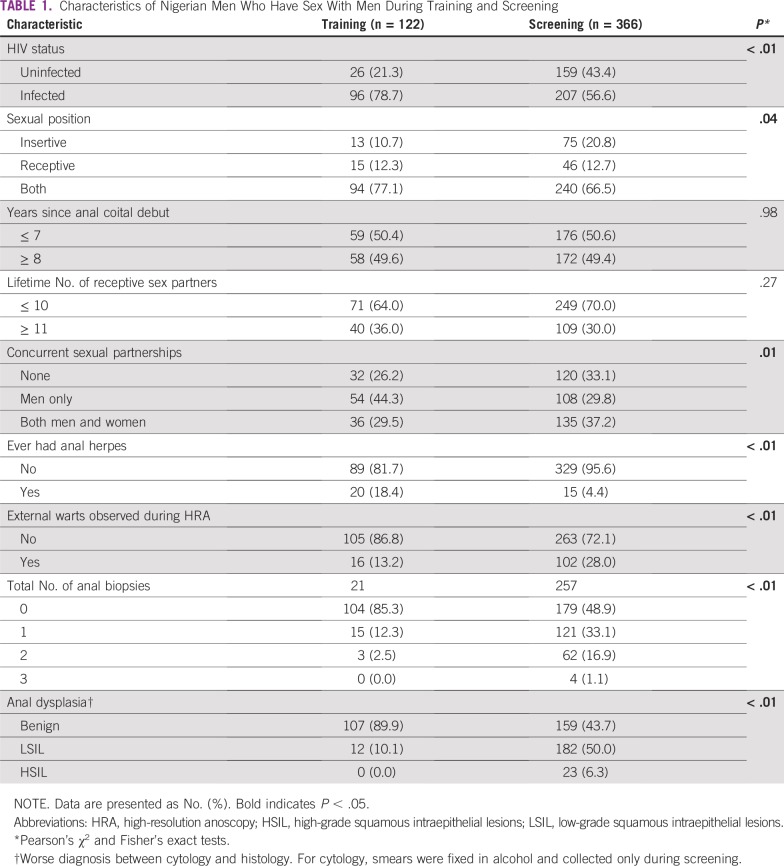
Characteristics of Nigerian Men Who Have Sex With Men During Training and Screening

In the crude analysis, a higher proportion of men with LSIL or HSIL had HIV, 8 or more years since anal coital debut, more lifetime partners, any concurrency, and external warts compared with those with a benign diagnosis. A higher proportion of men with HSIL had urethral STIs relative to men with a benign or LSIL diagnosis (Table 2). Five HSILs were detected among men with urethral STIs; all these men reported engaging in receptive sex. In the multivariable analysis, men had an increased odds of LSIL when they had HIV (*P* = .02), concurrent partnerships with men (*P* < .01), and external warts (*P* < .01). Urethral STIs (*P* = .01), external warts (*P* < .01), and an anal coital debut of more than 8 years (*P* = .04) increased the odds of HSIL (Table 3).

**TABLE 2:**
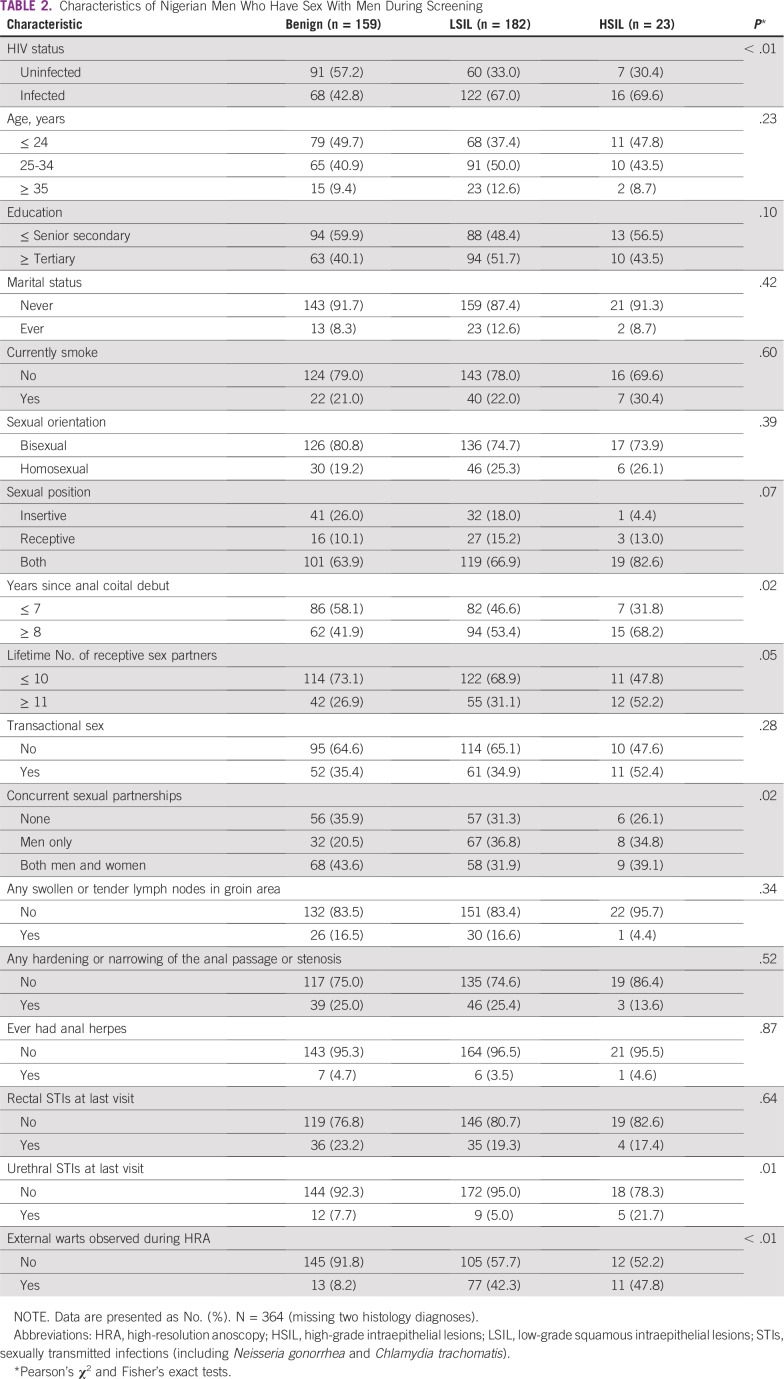
Characteristics of Nigerian Men Who Have Sex With Men During Screening

**TABLE 3:**
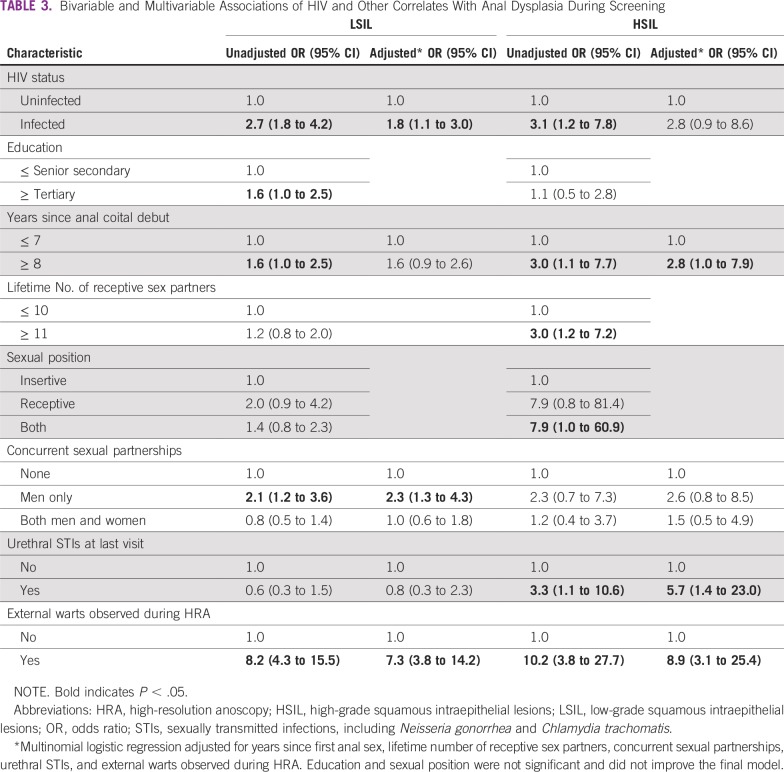
Bivariable and Multivariable Associations of HIV and Other Correlates With Anal Dysplasia During Screening

## DISCUSSION

Anal cancer screening using HRA as a first-line screening tool was well accepted, with 95% enrollment in the program among MSM attending a community-engaged health care facility in Abuja, Nigeria. Previous studies have reported on the challenge of conducting HRA^5-8^ and have recommended quality assurance metrics of 100 or more HRAs per year and identifying 50 or more cases of HSIL.^15^ Our team conducted more than 500 HRAs in 1 year, meeting the recommended volume of screenings, and diagnosed 23 cases of HSIL.^15^ To better understand our clinician’s level of proficiency given the low number of HSILs, we evaluated the prevalence of detecting any SIL over time.^6^ Similar to a prior study in the Netherlands,^6^ 200 screenings were needed before the clinician consistently diagnosed the same prevalence of any SIL over time.^6,8^ Our HSIL prevalence was consistent with that observed in some of the first studies of HRA^19,20^ but may become higher once the clinician achieves a similar S-shaped curve for HSIL detection. Monitoring diagnostic prevalence^6^ over time allows an assessment of clinician progress on the part of the learning curve where experience is being gained.

Another metric for evaluating clinical capacity is monitoring the proportion of men undergoing a biopsy. Hillman et al^15^ suggest performing more than one biopsy for each new patient depending on risk and prior screening experiences. Our biopsy rate was 15% during training, and 3% had more than one biopsy. Given the higher risk of HSIL observed during training, the low biopsy rate of the first 122 men indicates that some SIL may have been missed, confirming the time and volume needed to master HRA. The clinician’s biopsy rate increased to 51% during the pilot, but the multiple biopsy rate was less than 20%, and the detection of HSIL was low. Additional consultation with an experienced HRA clinician and the pathologist could improve the clinician’s technique. Another study reported a similar range of biopsy rates for different clinics, from 17% to 55%.^7^ In that study, the men were older, were HIV infected, and had many lifetime partners, and yet the study reported an HSIL prevalence of only 10%. If biopsy rates increase from 20% to 50%, then the clinic is improving, but it may be an indication that the study is yet to develop adept screening procedures as outlined by Hillman et al.^15^ Our HSIL prevalence of 6% with more training may rise to as high as 15%, as seen in a study population with a similar age,^21^ but not 20% to 40% as seen in older men.^22-26^ Biopsy rates are an additional indication of proficiency and improve the interpretation of reported prevalence estimates across studies.

Having external warts at the time of screening was the strongest independent risk factor for LSIL or HSIL. Warts may indicate receptive sexual practices, potential exposure to oncogenic HPV alongside wart-associated HPVs, or an immune system predisposed to poor clearance with higher risk of active or persistent HPV.^27^ Prior studies have found that the prevalence of anal warts and HSIL is much higher during HIV infection.^28-30^ In some cases, lesions that seem visually to be LSIL or benign warts also contain histologically defined HSIL.^31-33^ Genital warts are associated with an increased risk of anal cancer,^34-36^ and this risk is sustained even 10 years after diagnosis.^35^ Visualization of anal warts on physical examination may be considered an indication for health care providers to conduct HRA for anal cancer screening.

Our study enrolled a much younger population compared with other HRA clinics, but many of the men had been engaging in anal sex for enough time to develop HSIL. Having 8 or more years since anal coital debut was independently associated with the presence of HSIL and trended toward an association with LSIL (*P* = .08). Assuming that the natural history of HR-HPV is similar, this exposure time parallels the finding in women in which several years of persistent HR-HPV was associated with developing high-grade lesions.^37^ Some studies have evaluated age at coital debut as a precursor of HSIL, but it did not manage to demonstrate significant predictive value.^21,38^ Other studies evaluated age and found mostly a null relationship,^2,25,39^ whereas one reported a protective effect.^22^ Years since anal coital debut, instead of age, potentially indicates a person’s exposure history to HR-HPV and could be used by clinicians in an MSM-friendly clinic to identify men for screening.

Concurrent sexual partnerships with men only was an independent predictor of LSIL but not HSIL (*P* = .13). LSIL is an active, transient HPV infection that may be indicative of a recent exposure to HPV.^17^ HSIL, in contrast, is indicative of a persistent infection with HR-HPV.^17^ Therefore, recent sexual behavior, as seen with concurrency, may be more associated with LSIL than with HSIL. Consistent with our study, a study by Machalek et al^25^ found that recent receptive practices were independently associated with HSIL-AIN2 but not HSIL-AIN3. Because HSIL-AIN2 are equivocal lesions that fall between LSIL and HSIL, they may have an active transient component to their infection. More importantly, restricting analyses to focus only on HSIL^7,22,25^ limits our ability to disentangle factors that may be associated with either of these lesions, because they may represent different manifestations of HPV.

This study has some limitations. There was a low rate of diagnosis of HSIL. Many of the men had internal warts that potentially obstructed the view of the flatter lesions of HSIL.^33^ Earlier training in the treatment of internal warts may have helped improve the visibility and diagnosis of HSIL. In the quality assurance and control review of histologic diagnoses, the wide CIs of the Kappa statistic, resulting from the small sample size, limited its usefulness. Persistent HPV-16 confers the highest risk of HSIL,^25,40^ but testing for type-specific HPVs is ongoing. Finally, the risk factor analysis was cross-sectional and could not assess temporality.

Anal cancer screening was implemented successfully as a first-line screening tool in an MSM-friendly health care facility in Nigeria, although additional mentoring and time might have improved the quality of the HRA. HIV, concurrency, early coital debut, and external warts were independently associated with anal dysplasia. If anal cancer screening proves beneficial, these factors may help risk-stratify men.

## Data Availability

The following represents disclosure information provided by authors of this manuscript. All relationships are considered compensated. Relationships are self-held unless noted. I = Immediate Family Member, Inst = My Institution. Relationships may not relate to the subject matter of this manuscript. For more information about ASCO's conflict of interest policy, please refer to www.asco.org/rwc or ascopubs.org/jgo/site/misc/authors.html. **Honoraria:** BD Medical, Roche Molecular Diagnostics, Antiva, Boston Scientific/nVision **Consulting or Advisory Role:** BD Medical, Roche Molecular Diagnostics, Antiva, Boston Scientific/nVision **Consulting or Advisory Role:** American Gene Technologies, Scarab Genomics, Institute of Human Virology Nigeria **Patents, Royalties, Other Intellectual Property:** I am editor-in-chief of the *Journal of AIDS* **Stock and Other Ownership Interests:** Ubiome, Virion Therapeutics, VIR Biotechnology **Honoraria:** Janssen Pharmaceuticals, Vaccitech, Antiva Biosciences **Consulting or Advisory Role:** Antiva Biosciences, VIR Biotechnology, Vaccitech, Novan **Research Funding:** Merck (Inst), Antiva Biosciences (Inst), VIR Biotechnology (Inst), CEL-SCI (Inst) **Travel, Accommodations, Expenses:** Merck, Vaccitech, Janssen No other potential conflicts of interest were reported.

## APPENDIX

The TRUST Study Group is constituted as follows: **Principal Investigators:** Manhattan Charurat (IHV, University of Maryland, Baltimore, MD, USA), Julie Ake (MHRP, Walter Reed Army Institute of Research, Silver Spring, MD, USA); **Co-Investigators:** Sylvia Adebajo, Stefan Baral, Erik Billings, Trevor Crowell, George Eluwa, Charlotte Gaydos, Sosthenes Ketende, Afoke Kokogho, Hongjie Liu, Jennifer Malia, Olumide Makanjuola, Nelson Michael, Nicaise Ndembi, Jean Njab, Rebecca Nowak, Oluwasolape Olawore, Zahra Parker, Sheila Peel, Habib Ramadhani, Merlin Robb, Cristina Rodriguez-Hart, Eric Sanders-Buell, Sodsai Tovanabutra, Erik Volz; **Institutions:** Institute of Human Virology at the University of Maryland School of Medicine (IHV-UMB), University of Maryland School of Public Health (UMD SPH), Johns Hopkins Bloomberg School of Public Health (JHSPH), Johns Hopkins University School of Medicine (JHUSOM), U.S. Military HIV Research Program (MHRP), Walter Reed Army Institute of Research (WRAIR), Henry M. Jackson Foundation for the Advancement of Military Medicine (HJF), Henry M. Jackson Foundation Medical Research International (HJFMRI), Institute of Human Virology Nigeria (IHVN), International Centre for Advocacy for the Right to Health (ICARH), The Initiative for Equal Rights (TIERS), Population Council (Pop Council) Nigeria, Imperial College London.
